# Smoking Does Not Alter Treatment Effect of Intravenous Thrombolysis in Mild to Moderate Acute Ischemic Stroke—A Dutch String-of-Pearls Institute (PSI) Stroke Study

**DOI:** 10.3389/fneur.2020.00786

**Published:** 2020-07-31

**Authors:** Anna Kufner, Martin Ebinger, Gert Jan Luijckx, Matthias Endres, Bob Siegerink

**Affiliations:** ^1^Center for Stroke Research Berlin (CSB), Department of Neurology, Charité Universitätsmedizin Berlin, Berlin, Germany; ^2^Berlin Institute of Health (BIH), Berlin, Germany; ^3^Klinik für Neurologie, Charité Universitätsmedizin Berlin, Berlin, Germany; ^4^Department of Neurology, Medical Park Berlin Humboldtmühle, Berlin, Germany; ^5^Department of Neurology, University Medical Centre Groningen, University of Groningen, Groningen, Netherlands; ^6^German Centre for Cardiovascular Research (DZHK), Berlin, Germany; ^7^German Center for Neurodegenerative Diseases (DZNE), Berlin, Germany; ^8^Excellence Cluster NeuroCure, Charite-Universitätsmedizin Berlin, Berlin, Germany

**Keywords:** stroke, smoking, thrombolysis (tPA), ischemic stroke, cerebrovascular risk factors

## Abstract

**Background:** The smoking-thrombolysis paradox refers to a better outcome in smokers who suffer from acute ischemic stroke (AIS) following treatment with thrombolysis. However, studies on this subject have yielded contradictory results and an interaction analysis of exposure to smoking and thrombolysis in a large, multicenter database is lacking.

**Methods:** Consecutive AIS patients admitted within 12 h of symptom onset between 2009 and 2014 from the prospective, multicenter stroke registry (Dutch String-of-Pearls Stroke Study) were included for this analysis. We performed a generalized linear model for functional outcome 3 months post-stroke depending on risk of the exposure variables (smoking yes/no, thrombolysis yes/no). The following confounders were adjusted for: age, smoking, hypertension, atrial fibrillation, diabetes mellitus, stroke severity, and stroke etiology.

**Results:** Out of 468 patients, 30.6% (*N* = 143) were smokers and median baseline NIHSS was 3 (interquartile range 1–6). Smoking alone had a crude and adjusted relative risk (RR) of 0.99 (95% CI 0.89–1.10) and 0.96 (95% CI 0.86–1.01) for good outcome (modified Rankin Score ≤ 2), respectively. A combination of exposure variables (smoking and thrombolysis) did not change the results significantly [crude RR 0.87 (95% CI 0.74–1.03], adjusted RR 1.1 (95%CI 0.90–1.30)]. Smoking alone had an adjusted RR of 1.2 (95% CI 0.6–2.7) for recanalization following thrombolysis (*N* = 88).

**Conclusions:** In patients with mild to moderate AIS admitted within 12 h of symptom onset, smoking did not modify treatment effect of thrombolysis.

## Introduction

The so-called smoking-paradox of an improved outcome following thrombolysis was first described in smokers with myocardial infarction (1, 2). This phenomenon has resurfaced as a topic of interest in the scientific community as a handful of recent studies have reported similar observations in acute ischemic stroke patients treated with recombinant tissue plasminogen activator (r-tPA) as well as endovascular therapies (3–6).

The mechanism underlying the pathophysiology of the smoking-thrombolysis paradox remains unclear. Some attribute the observed phenomenon to a systematic lack of adjustment of confounding factors i.e., lower clinical risk profiles of smokers due to younger age and fewer comorbidities (7, 8). Others argue that there is substantial evidence supporting an alteration of clot dynamics (9, 10) caused by smoke exposure leading to enhanced tPA efficacy in patients with this risk factor.

As of yet, the studies on this subject have yielded contradictory results (3–8, 11, 12). Most likely, there is a cumulative effect of younger age, lower clinical risk profiles, and more aggressive treatment effect that account for the smoking-thrombolysis paradox. However, a large comprehensive interaction analysis of exposure to smoking and treatment with thrombolysis is still lacking. Therefore, we set out to determine the measures of interaction of smoking status (current smokers vs. non-smokers) and treatment (thrombolysis vs. no thrombolysis) and their attributable risk in terms of functional recovery 3 month post-stroke and recanalization rates in a large, national multicenter stroke registry.

## Methods

### Patients

All data comes from the Dutch String-of-Pearls Stroke Study—a prospective, multicenter cohort study in which patients with recent stroke presenting within 1-month after symptom onset are eligible for enrollment following informed consent (13). Local ethics committees of all participating hospitals approved the study and all patients provided written informed consent. Research with this data was performed in accordance with the Medical Research Involving Human Subjects Act and codes developed by the Dutch Federation of Medical Scientific Societies.

**Figure 1:**
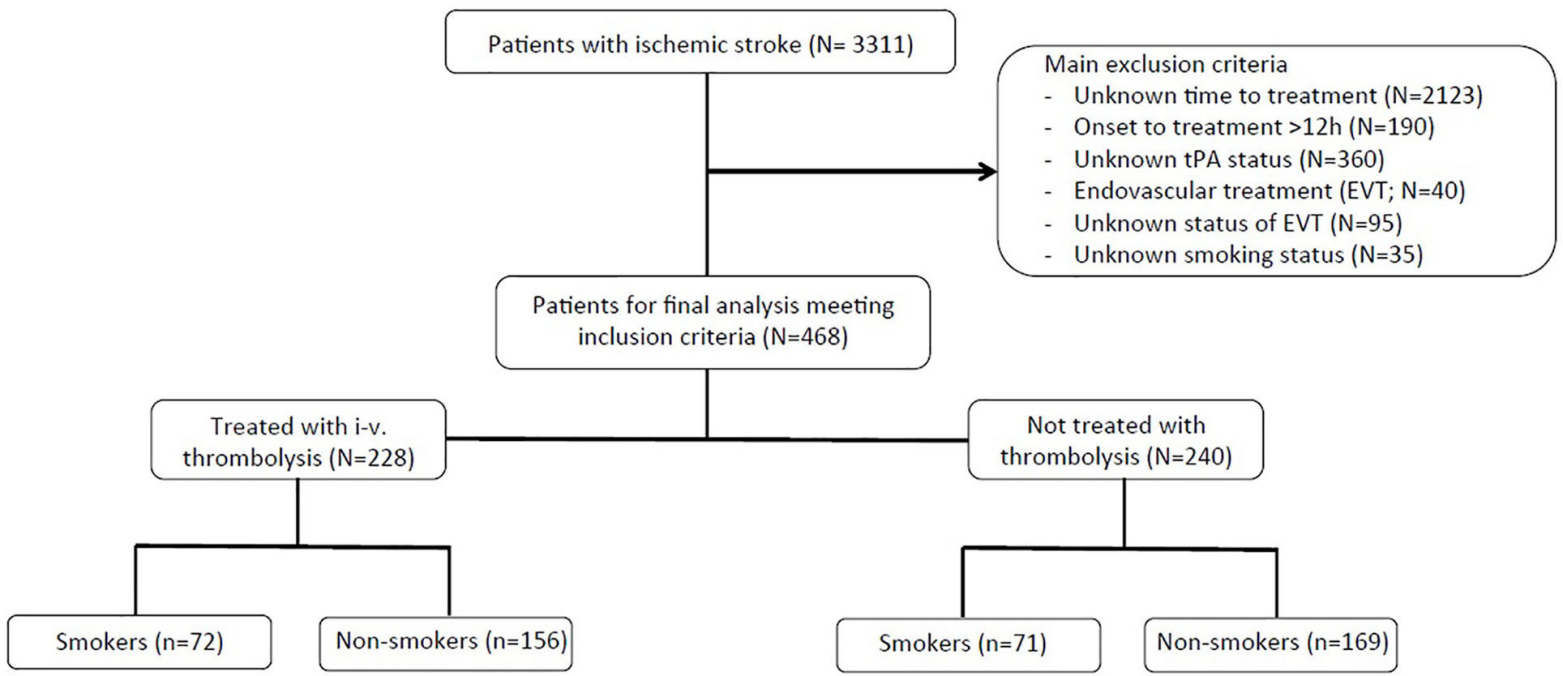
Flow-chart diagram illustrating patient selection for current analyses.

Consecutive acute ischemic stroke patients between October 2009 and October 2014 were included for this analysis. Major inclusion criteria for the current study include the following: ischemic stroke determined via CT or MRI, known time of symptom onset, admission within 12 h of symptom onset, and documented smoking status at the time of the event. Patients were excluded from the analysis if they received endovascular therapies or if endovascular treatment status was unknown or undocumented. A flow diagram shows the patient selection criteria for this sub-study (Figure 1).

### Regression Analyses

We performed regression analyses for binary endpoints by generalized linear model (glm) using a modified log-Poisson regression model with a robust error variance to reduce risk of overestimation (14). These models were used to estimate relative risks for good functional outcome (mRS ≤ 2), excellent functional outcome (mRS dichotomized at ≤ 1), and mortality 3 months post-stroke depending on risk of the exposure variables (smoking yes/no, thrombolysis yes/no). The two aforementioned exposure variables create four patient groups with combined exposures of smoking and treatment (i.e., –/–, −/+, +/–, and +/+). Crude and adjusted relative risk (RRs) were calculated for the four patient groups based on the exposures. Models were adjusted for age, hypertension, diabetes mellitus, atrial fibrillation, stroke severity (NIHSS), and stroke etiology.

## Results

### Patient Characteristics

Four hundred sixty-eight patients were included in the final analysis; 30.6% were smokers. For a detailed description of basic demographics and baseline parameters of the entire study group, as well as sub-groups based on smoking-status, refer to Table 1. Compared to non-smokers, smokers had lower rates of hypertension, and atrial fibrillation. Stroke etiology differed significantly between groups; smokers presented with higher rates of large-artery atherosclerotic strokes compared to non-smokers who presented with higher rates of cardioembolic stroke. Infarct localization and stroke severity did not differ among groups (Table 1).

**Table 1:** Basic demographics and baseline clinical parameters of the entire cohort, and sub-groups based on smoking status.

	**All patients (*N* = 468)**	**Smokers (*N* = 143)**	**Non-smokers (*N* = 325)**
Age, mean (*SD*)	66.2 (13.8)	58.9 (12.7)	69.3 (13.0)
Sex, *n* (%) female	206 (44.0)	63 (44.1)	143 (44.0)
**Cerebrovascular risk factors**, ***n*** **(%)**
Diabetes mellitus	78 (16.8)	17(12.1)	61 (18.8)
Hyperlipidemia	119 (26.1)	34 (24.1)	85 (27.0)
Atrial fibrillation	56 (12.1)	9 (6.4)	47 (14.6)
Hypertension	231 (50.1)	58 (40.9)	173 (54.2)
Baseline systolic blood pressure, median mmHg (IQRL)	157 (138–175)	153 (137–178)	157.5 (140–173)
Body weight in kilograms, median IQRL	79 (67–87.5)	80 (69–88)	75 (64–87)
Baseline NIHSS, median (IQRL)	3 (1–6)	3 (1–7)	2 (1–6)
Time to treatment, median (IQRL)	105 (75–150)	100 (72–143)	110 (75–155)
Thrombolysis, *n* (%)	228 (48.7)	72 (50.3)	156 (48.0)
**Infarction in arterial territories**, ***n*** **(%)**
ACA	173 (39.1)	55 (41.0)	118 (38.2)
PCA	32 (7.3)	6 (4.5)	26 (8.4)
MCA	105 (23.5)	36 (26.5)	69 (22.2)
Basilar artery	40 (9.0)	13 (9.8)	27 (8.7)
Vertebral artery	20 (4.5)	2 (1.5)	18 (5.8)
**Stroke etiology (TOAST)**, ***n*** **(%)**
Large-artery atherosclerosis	105 (23.5)	33 (24.8)	72 (22.9)
Cardioembolism	88 (23.5)	15 (11.3)	73 (23.3)
Small vessel occlusion	85 (19.7)	36 (27.1)	49 (15.6)
Other	30 (6.7)	10 (7.5)	20 (6.4)
Undetermined	139 (31.1)	39 (29.3)	100 (31.9)

*All variables had <5% missing, with TOAST having the largest percentage of missings (i.e., 4.5%). SD, standard deviation; NIHSS, National Institute of Stroke Scale; IQRL, interquartile range limit; ACA, anterior cerebral artery; PCA, posterior cerebral artery; MCA, middle cerebral artery; TOAST, Trial of Org 10172 in Acute Stroke Treatment*.

### Generalized Linear Model for Functional Recovery and Recanalization

In the entire cohort (*N* = 468), comparing smokers and non-smokers revealed no significant differences in terms of good outcome of mRS ≤ 2 (70.6 vs. 72.9%), excellent outcome of mRS ≤ 1 (46.2 vs. 55.4%), or mortality (4.2 vs. 4.9%). In sub-group analysis including only patients treated with thrombolysis (*N* = 228; Table 2), results were similar; we observed no differences in univariate analysis between smokers and non-smokers for any of the primary outcome endpoints. Overall rate of hemorrhagic transformation following thrombolysis was 3.1%; rates did not differ significantly between groups.

**Table 2:** Outcome parameters assessed 3 months post-stroke (good outcome [mRS ≤ 2], excellent outcome [mRS ≤ 1], and mortality), as well as rates of recanalization and hemorrhagic transformation reported in all patients who received intravenous thrombolysis (*N* = 228) and based on smoking status.

	**All patients (*N* = 228)**	**Smokers (*N* = 72)**	**Non-smokers (*N* = 156)**	**Difference smokers—non-smokers (95% CI)**
Good outcome at 3 months, *n* (%)	147 (71.4)	46 (73.0)	101 (70.6)	0.024 (−0.11 to 0.16)
Excellent outcome at 3 months, *n* (%)	102 (49.5)	27 (42.9)	75 (52.5)	−0.96 (−0.25 to 0.05)
Mortality at 3months, *n* (%)	10 (4.9)	2 (3.2)	8 (5.6)	−0.02 (−0.08 to 0.03)
Recanalization, *n* (%)	22 (25)	9 (29.0)	13 (22.8)	0.06 (−0.14 to 0.26)
Hemorrhagic transformation, *n* (%)	7 (3.1)	1 (1.4)	6 (3.8)	−0.03 (−0.08 to 0.02)

Smoking status had a crude and adjusted RR of 0.99 (95% CI 0.89–1.11) and 0.96 (95% CI 0.86–1.01) for a good outcome, respectively. In a sub-group analysis including only patients with documented recanalization status (*N* = 88), smoking had a crude RR of 1.3 (95% CI 0.6–2.7), and an adjusted RR (including age, time-to-treatment, stroke etiology) of 1.2 (95% CI 0.6–2.7) for recanalization. Results from the combined exposure analyses (i.e., Smoking + thrombolysis) for a good outcome (mRS ≤ 2), excellent outcome (mRS ≤ 1), and mortality 3 months following the index event are presented in Table 3. In short, no clear interaction could be observed.

**Table 3:** Relative risks (RR) for a good outcome (modified Rankin Score [mRS] ≤ 2), excellent outcome (mRS ≤ 1), and mortality 3 months post-stroke, presenting crude, and adjusted values (adjusted for age, presence of hypertension, diabetes mellitus, atrial fibrillation, National Institute of Stroke Scale on admission, stroke etiology categories).

**Thrombolysis**	**Smoking**	**Number of endpoints/total**	**Crude RR (95% CI)**	**Adjusted RR (95% CI)**
**GOOD OUTCOME mRS** **≤** **2**				
–	–	136/162	1 (reference)	1 (reference)
–	+	55/68	0.96 (0.84–1.10)	0.93 (0.82–1.05)
+	–	101/143	0.84 (0.74–0.95)	1.06 (0.94–1.20)
+	+	46/63	0.87 (0.74–01.03)	1.08 (0.90–1.30)
**EXCELLENT OUTCOME mRS** **≤** **1**				
–	–	105/162	1 (reference)	1 (reference)
–	+	39/68	0.88 (0.70–1.12)	0.88 (0.70–1.11)
+	–	75/143	0.81 (0.67–0.98)	1.11 (0.91–1.37)
+	+	27/63	0.66 (0.49–0.90)	0.91 (0.65–1.27)
**MORTALITY**				
–	–	8/162	1 (reference)	–
–	+	4/68	1.29 (0.4–3.8)	–
+	–	8/143	1.1 (0.4–2.9)	–
+	+	2/63	0.6 (0.1–3.0)	–

*Adjustment was not performed for mortality analyses due to low numbers*.

## Discussion

We observed no biological interaction of smoking and intravenous thrombolysis in terms of functional recovery 3 months post-stroke in this multicenter cohort of mild to moderate ischemic stroke patients admitted within 12 h of symptom onset.

In all patients, and in sub-group analysis including only those treated with intravenous thrombolysis, there was no difference in functional outcome between smokers and non-smokers in univariate analysis (Table 2). Interestingly, both crude and adjusted analyses suggest that smoking alone has no effect on long-term functional recovery post-stroke despite lower clinical risk profiles of these patients, although the precision of these analyses is limited. In our combined exposure analyses, smoking alone had a crude and adjusted RR of 0.88 for an excellent outcome. The seemingly adverse effect of tPA-administration alone on functional recovery disappeared when confounding factors such as stroke severity were considered (crude RR of 0.84 and adjusted RR of 1.06 compared to crude RR of 0.81 and adjusted RR 1.11 for good and excellent outcome, respectively), which can be explained by confounding by indication. A combination of tPA-administration and smoking did not lead to an improved functional outcome (Table 3). These results are an indicator that smoking status negatively influences outcome, regardless of treatment with thrombolysis in this cohort.

Similar to previous reports (3–5, 8), ~31% of patients were smokers. As expected, patients with this risk factor had fewer cerebrovascular comorbidities [hypertension (3, 4, 11) and AF (11)], and more frequently suffered from large-artery atherosclerotic strokes (4, 11) (Table 1). Smoking led to increased recanalization rates in patients treated with thrombolysis in this cohort [adjusted RR 1.2 (95% CI 0.6–2.7)]. However, due to small numbers an additive interaction analysis for smoking and thrombolysis for recanalization could not be performed and results should be interpreted with caution.

This cohort included predominately minor strokes (median baseline NIHSS: 3 IQR 1–6). Assuming smoking may modify treatment effect of thrombolysis by increasing recanalization rates of large vessel occlusions, any biological interaction of smoking, and tPA may have been missed in this analysis. Previous studies that found an enhanced treatment efficacy in smokers included patients with more severe strokes and higher rates of proven vessel occlusion (3–6). Therefore, a similar analysis in an independent cohort of patients with large vessel occlusion is warranted to further shed light on a possible increased treatment efficacy of thrombolysis in smokers.

An important limitation of this study is the exclusion of a large number of patients with undocumented time to treatment. This is most likely due to the fact that this stroke registry consists of subacute stroke patients with onset within 30 days, PSI where time to treatment has less clinical relevancy. This certainly led to a selection of patients which limits extrapolation of our results into other populations, notably to those without known stroke onset. An additional limitation of this analysis is missing information relevant to our research question (i.e., vessel occlusion size and methods applied for assessment of recanalization) as well as missing data on pre-treatment glucose levels and rates of symptomatic intracerebral hemorrhage which may have influenced functional outcome in these patients (15). These limitations are inherent to our study design, as data was retrospectively analyzed from a stroke registry not primarily designed to address our research aim.

In conclusion, smoking did not modify treatment effect of thrombolysis in acute ischemic stroke patients in this cohort. However, a large, multicenter cohort analysis including patients with proven vessel occlusion and more severe strokes is warranted to further investigate a potential biological interaction between smoking and intravenous and intra-arterial thrombolysis.

## Data Availability Statement

The data analyzed in this study was subject to the following licenses/restrictions: data will be made available upon reasonable request. Requests to access these datasets should be directed to bob.siegerink@charite.de.

## Ethics Statement

The studies involving human participants were reviewed and approved by the String-of-Pearls Institute has provided a regulatory framework in which ethical and legal rules and guidelines have been described (http://www.string-of-pearls.org/). The patients/participants provided their written informed consent to participate in this study.

## Author Contributions

AK, MEb, MEn, and BS conceived and designed the project. AK and BS performed the data analysis. AK wrote the first draft of the manuscript. All authors interpreted the data, reviewed and edited the manuscript, and approved the final version of the manuscript.

## Conflict of Interest

The authors declare that the research was conducted in the absence of any commercial or financial relationships that could be construed as a potential conflict of interest.
